# Recent advances in drug delivery systems for targeting brain tumors

**DOI:** 10.1080/10717544.2022.2154409

**Published:** 2023-01-03

**Authors:** Yi Zhao, Ping Yue, Yao Peng, Yuanyuan Sun, Xing Chen, Ze Zhao, Bingjie Han

**Affiliations:** a Department of Translational Medicine Center, the First Affiliated Hospital of Zhengzhou University, Zhengzhou, China; b The Academy of Medical Science, College of Medical, Zhengzhou University, Zhengzhou, China; c Key Laboratory of Drug-Targeting and Drug Delivery System of the Education Ministry and Sichuan Province, Sichuan Engineering Laboratory for Plant-Sourced Drug and Sichuan Research Center for Drug Precision Industrial Technology, West China School of Pharmacy, Sichuan University, Chengdu, China; d Department of Orthopedics, the First Affiliated Hospital of Henan Polytechnic University (the Second People’s Hospital of Jiaozuo City), Jiaozuo, China

**Keywords:** Nano drug delivery system, brain tumor, nanoparticle, brain targeting, combination therapy

## Abstract

Brain tumor accounts for about 1.6% of incidence and 2.5% of mortality of all tumors, and the median survival for brain tumor patients is only about 20 months. The treatment for brain tumor still faces many challenges, such as the blood-brain barrier (BBB), blood-brain tumor barrier (BBTB), the overexpressed efflux pumps, the infiltration, invasion, high heterogeneity of tumor cells, drug resistance and immune escape caused by tumor microenvironment (TME) and cancer stem cells (CSC). This review attempts to clarify the challenges for multi-functional nano drug delivery systems (NDDS) to cross the BBB and target the cancer cells or organelles, and also provides a brief description of the different types of targeted multi-functional NDDS that have shown potential for success in delivering drugs to the brain. Further, this review also summarizes the research progress of multi-functional NDDS in the combination therapy of brain tumors from the following sections, the combination of chemotherapy drugs, chemotherapy-chemodynamic combination therapy, chemotherapy-immunization combination therapy, and chemotherapy-gene combination therapy. We also provide an insight into the recent advances in designing multi-functional NDDS for combination therapy.

## 1. Introduction

Brain tumor accounts for about 1.6% of incidence and 2.5% of mortality of all tumors respectively according to the latest global cancer data released by the World Health Organization (WHO) in 2020 (Sung et al., 2021). While, in China, the morbidity and mortality of brain tumors rank first in the global brain tumors, up to 32% and 26% respectively, and the incidence rate is still rising year by year and younger (Patel et al., 2019). Among the brain tumors, glioma, the most common and invasive type of brain tumor, with the characteristics of strong invasion, high recurrence rate and poor prognosis, accounts for 30% of all brain tumors (Reifenberger et al., 2017; Lin et al., 2020).

Surgery is the treatment of choice for brain tumors, but the invasiveness and fuzzy boundary make it difficult to completely remove the tumor. And, the postoperative recurrence rate is more than 90% (Ganz, 2022). Moreover, postoperative chemotherapy and radiotherapy have become the standard therapy for brain tumors. An alkylating agent, temozolomide (TMZ), functions as a first-line chemotherapy drug for brain tumors and delivers the methyl group to purine bases of DNA to cause cell death (Zhang et al., 2012). However, the increased dosage due to its short half-life, which leading to a series of side effects, such as thrombocytopenia, neutropenia and lymphopenia. In addition, the tumor cells may become resistant to TMZ due to the dysregulation of signaling pathways, DNA repair, autophagy and other related mechanisms (Yan et al., 2016). Apart from TMZ, bevacizumab has been approved by USA FDA for the treatment of brain tumors as a VEGFR inhibitor. However, this anti-angiogenesis therapy has failed to improve the overall survival of patients, and its use remains controversial (Ozdemir-Kaynak et al., 2018). Other therapeutic drugs, such as nitrosoureas (carmustine, lomustine), anthracyclines (adriamycin), platinums (cisplatin, carboplatin, oxaliplatin), topoisomerase inhibitors (camptothecin, irinotecan, etoposide), integrin receptor inhibitors (cilengitide), EGFR inhibitors (erlotinib, gefitinib, afatinib), and histone deacetylase inhibitors (vorinostat, panobinostat), are difficult to become specific drugs for the treatment of brain tumors due to the low efficacy and severe toxic and side effects (Aparicio-Blanco et al., 2020). New therapies such as gene therapy, angiogenesis inhibition and immunotherapy have shown potential but limited efficacy in the treatment of glioma (Sousa et al., 2019; Weenink et al., 2020; Chelliah et al., 2021; Conniot et al., 2021). Therefore, there is an urgent need to develop high-efficiency, low-toxicity and specific drugs for brain tumors.

## 2. Challenges in developing drugs for brain cancer

**Figure 1.**
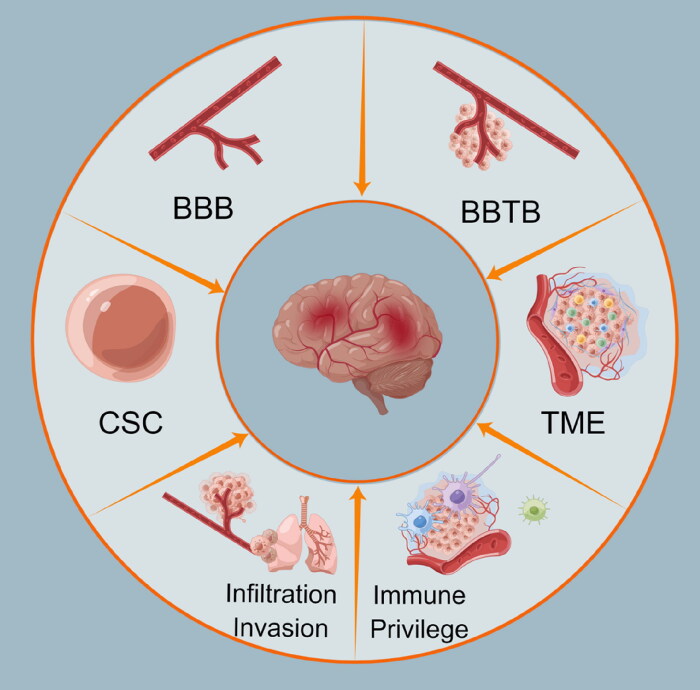
Challenges in treatment for brain cancer (Drawn by Figdraw).

Compared with peripheral tumors, the treatment of brain tumors faces many challenges (Figure 1). On the one hand, the physiological barriers (such as blood-brain barrier (BBB), blood-brain tumor barrier (BBTB)) and the over-expressed efflux pumps prevent drugs from entering the central nervous system (CNS) and reaching the tumor site. On the other hand, the inherent characteristics of brain tumors, such as the infiltration, invasion, high heterogeneity, drug resistance and immune escape caused by tumor microenvironment (TME) and cancer stem cells (CSC), further restrict the therapeutic effects, which leading to high failure rate and recurrence rate (Zhao et al., 2020). The median survival of brain tumor patients receiving standard therapy is only about 20 months, and the 2- and 5-year survival rates are only 27% and 10%, respectively (Ashby et al., 2016).

### 2.1. Blood-brain barrier and blood-brain tumor barrier

The BBB consists of brain capillary endothelial cells (BCEC), pericytes, astrocytes, neurons, and basement membranes, and it plays a crucial role in the transport of endogenous and exogenous molecules between the blood and the brain (Zhao et al., 2022). The BBB prevents all the macromolecular drugs and over 98% of small molecule drugs to permeate into CNS based on the following mechanisms. (1) Paracellular barrier: The tight junctions between BCEC cells strictly limit the passive diffusion of drugs into CNS, and only lipophilic substances and hydrophilic small molecules are allowed to enter the brain. (2) Transcellular barrier: The endocytosis activity is lower in BCEC cells than that in other brain cells, which significantly limits the transcellular transport of drugs across the BBB (Azarmi et al., 2020). (3) Enzyme barrier: The BBB cells have a strong metabolic capacity due to the significant high expression of peptidase, phosphatase, nucleotidase, esterase, and cytochrome P450 enzymes in BCEC, which enhances the ability to degrade drugs (Alexander, 2018). (4) Immunologic barrier: The microglia, mastocyte, and macrophages form an immunologic barrier to accelerate the clearance of drugs (Alexander, 2018). (5) Efflux proteins: The efflux proteins, such as ATP-binding cassette transporters (P-gp, BCRP, MRPs) and solute carrier transporters, are over-expressed at the BBB that actively pump out the drugs and limit the permeability (Saidijam et al., 2018). And it is also one of the main reasons for drug resistance in brain tumors.

When the brain tumor becomes larger than 2 mm^3^, the progression of angiogenesis results in the loss of normal functions and integrity of the BBB, and the BBTB comes into being (Mojarad-Jabali et al., 2021). Passive tumor targeting via enhanced permeability and retention (EPR) effect has long been considered as the most effective mechanism for the accumulation of nanoparticles. Whereas, the vascular pore size of brain tumors is much smaller (only 7 ∼ 100 nm) and the EPR is much weaker. Therefore, it is still difficult for drugs to reach brain tumor sites through the EPR effect (Caro et al., 2021). Hence, BBTB is considered to be another major obstacle for the drug transport in the treatment of brain tumors, critically restricting the delivery of drugs to tumor tissues.

### 2.2. Infiltration and invasion

The brain tumor cells show an aggressive characteristic against the surrounding tissues. Even individual brain tumor cell can infiltrate normal tissues and form tumors through the following steps. The brain tumor cells migrate and accumulate at the nearby vessels, and secrete the glioma-derived factors, such as TGF-β2, reactive oxygen species (ROS), and proinflammatory peptides that disrupt the normal contact between endothelial cells and the basement membrane. Subsequently, the factors induce and activate matrix metalloproteinases (MMPs), which further induce the degradation of tight junctions by downregulating claudin proteins. These processes contribute to the degradation of the vascular basement membrane and extracellular matrix, migration of endothelial cells, and the formation of abnormal new blood vessels due to VEGF overexpression (Ishihara et al., 2008; Dubois et al., 2014; Oishi et al., 2022). Therefore, the abnormally rapid proliferation of the vasculature leads to the function loss of tight junctions, i.e. the destruction of the BBB, which conduces to the infiltrative growth of the tumor with blurred tumor margins and metastasis.

### 2.3. Brain cancer stem cells

The cell subsets in brain tumors show stem cell-like characteristics and express stem cell markers, including CD133, A2B5 and EGFRvIII (Emlet et al., 2014; Ishii et al., 2021; Smiley et al., 2021). The stem cells exhibit the following characteristics. (1) Aggressiveness: Including the highly migratory and invasive, and the resistance to chemoradiotherapy. (2) Similar to normal stem cells or progenitor cells, the CSC can self-renew and differentiate into different types of cancer cell lines in specific tumor tissues. (3) Drug resistance: The multidrug resistance (MDR) of CSC is embodied in repairing DNA damage and excreting harmful substances (Phi et al., 2018). In addition, CSC can also enhance the transcription of anti-apoptotic genes and efflux transporters, and the angiogenesis. Although standard therapy kills most tumor cells, stem cells that have invaded the brain parenchyma will eventually lead to disease recurrence due to the invasiveness, resistance, self-renewal, and differentiation (Alcantara Llaguno & Parada, 2021). Therefore, eradicating tumor stem cells is an important research field to overcome MDR and improve the efficiency of tumor treatment.

### 2.4. Immune escape

The BBB prevents the entry of most harmful components, leaving the brain in a relatively safe environment and rarely launching immune attacks. Once the brain cells are attacked by autoimmunity, the consequences are serious. Therefore, the immune system of CNS is usually inhibited. On the other hand, the dogma has been established that the CNS lacks normal running lymphatic and dendritic cells for antigen presentation (D'Agostino et al., 2012). In consequence, active immune surveillance in the CNS rarely occurs, which provides a safe environment for tumor growth (Rustenhoven & Kipnis, 2019). Immunotherapy has been proved to have therapeutic potential for various solid tumors, including melanoma and non-small cell lung cancer (Waldman et al., 2020). However, the current immunotherapy has not been confirmed to significantly improve the survival rate of patients with brain tumors in clinic. It is mainly because the immune components such as antibodies and immune cells cannot enter the CNS through BBB (Desbaillets & Hottinger, 2021).

### 2.5. Tumor microenvironment

The tumor microenvironment includes tumor cells, tumor stem cells, blood vessels, lymphatic, immune cells, fibroblasts, and extracellular matrix, which provides a suitable environment for the growth, division, angiogenesis and metastasis of tumor cells (Petrova et al., 2018). And TME protects tumor cells mainly through the following mechanisms. The increased activity of vascular endothelial growth factor leads to the high proliferation of microvessels. Tumor cells interact with secreted cytokines or growth factors to obtain nutrients from abnormal blood vessels, which in turn induce fibroblasts and macrophages to proliferate and invade, resulting in drug resistance. The cross-linking structure of extracellular matrix formed by the fibrous collagen, proteoglycan, stromal cell protein and hyaluronic acid prevents drugs from reaching tumor cells through the microenvironment, thus resisting the drugs treatment. In addition to providing integral structure, extracellular matrix also contributes to the transport of nutrients and oxygen, thereby promoting tumor initiation and progression.

## 3. Design strategy of brain tumor targeting nano drug delivery system

Nano drug delivery system has unique advantages in drug delivery. The appropriate physicochemical properties including solubility, particle size, potential, and morphology contribute to improving the pharmacokinetics and tissue distribution. What’s more, surface modification may enhance the accumulation of drugs in the target tissue to improve the therapeutic effect. In addition, the NDDS has specific drug release behavior, which increases the concentration of drug in the target site and reduces the concentration of drugs in the non-target site, thereby reducing adverse reactions. Furthermore, the NDDS is easy to realize the combined treatment to achieve synergistic effects. Therefore, NDDS provides an excellent platform for the study of brain tumor-targeted drugs (Yeini et al., 2021). The commonly used design strategies are optimizing the physicochemical properties, overcoming the BBB and BBTB, introducing stimulus-responsive functional groups, and targeting organelles, et al.

### 3.1. Optimizing the physicochemical properties

The size, surface charge, morphology, and surface modification of nanoparticles influence the drug circulation in the blood and accumulation in the brain, which should be taken into consideration when designing NDDS.

Nanocarriers preferentially accumulate in tumor through passive targeting due to the leaky vasculature and defective lymphatic drainage (Subhan et al., 2021). Nanostructures smaller than 10 nm are rapidly cleared by the kidney, while larger than 200 nm are easily cleared by the liver and recognized by the reticuloendothelial system (RES) to reduce the circulation time in the blood (Kibria et al., 2013; Golombek et al., 2018). In addition, the vascular aperture of brain tumors is only 7 ∼ 100 nm, which is much smaller than that of other tumors. Moreover, the EPR effect is also much weaker than that of peripheral tumors. Therefore, the particle size of 10 ∼ 100 nm seems to be more efficient in crossing the BBB and delivering drugs into the brain.

The charge of nanoparticles has an effect on the interaction between nanomaterials and cells (Sanita et al., 2020). Compared with neutral nanoparticles, the charged nanoparticles have the following advantages. (1) High stability: Due to the lack of electrostatic interaction, the neutral nanoparticles have low physical stability and can’t inhibit the self-aggregation of nanoparticles. Meanwhile, the charges on the surface prevent nanoparticles from polymerizing and flocculating by zeta electrostatic interaction. (2) High permeability: Nanoparticles can interact with cells through surface electrostatic charges, so as to improve the accumulation of drugs in cells (Smith et al., 2017). For example, cationic nanoparticles interact with negatively charged BBB and are transported into the brain (Lombardo et al., 2020). However, cationic nanoparticles are easy to be rapidly cleared by RES and the positive surface charge leads to systemic adverse effects.

The morphology of nanoparticles also influences on the distributions. As reported, the cell uptake of rod-shaped particles (larger than 100 nm) is superior to spherical, cylindrical and cubic nanoparticles (Jia et al., 2021). However, spherical nanoparticles show the highest absorption when the diameter is less than 100 nm (Qiu et al., 2010).

The interaction between nanoparticles and biological microenvironment is an important factor to influence the fate of particles in vivo. This interaction depends not only on the physicochemical properties, but also on the surface modification and biomolecules in the biological environment. Qie et al. prepare the nanoparticles coated with polyethylene glycol (PEG) and CD47 to avoid the phagocytosis by macrophages (Qie et al., 2016). Although the PEGylation reduces the capacity of nanoparticles to adsorb a variety of soluble proteins, the immunogenicity is a potential limiting factor that may lead to the increased clearance rate and decreased efficacy of PEGylated nanoparticles upon repeated administration, which is known as accelerated blood clearance (ABC) phenomenon (Mohamed et al., 2019).

### 3.2. Overcoming the BBB and BBTB

**Figure 2.**
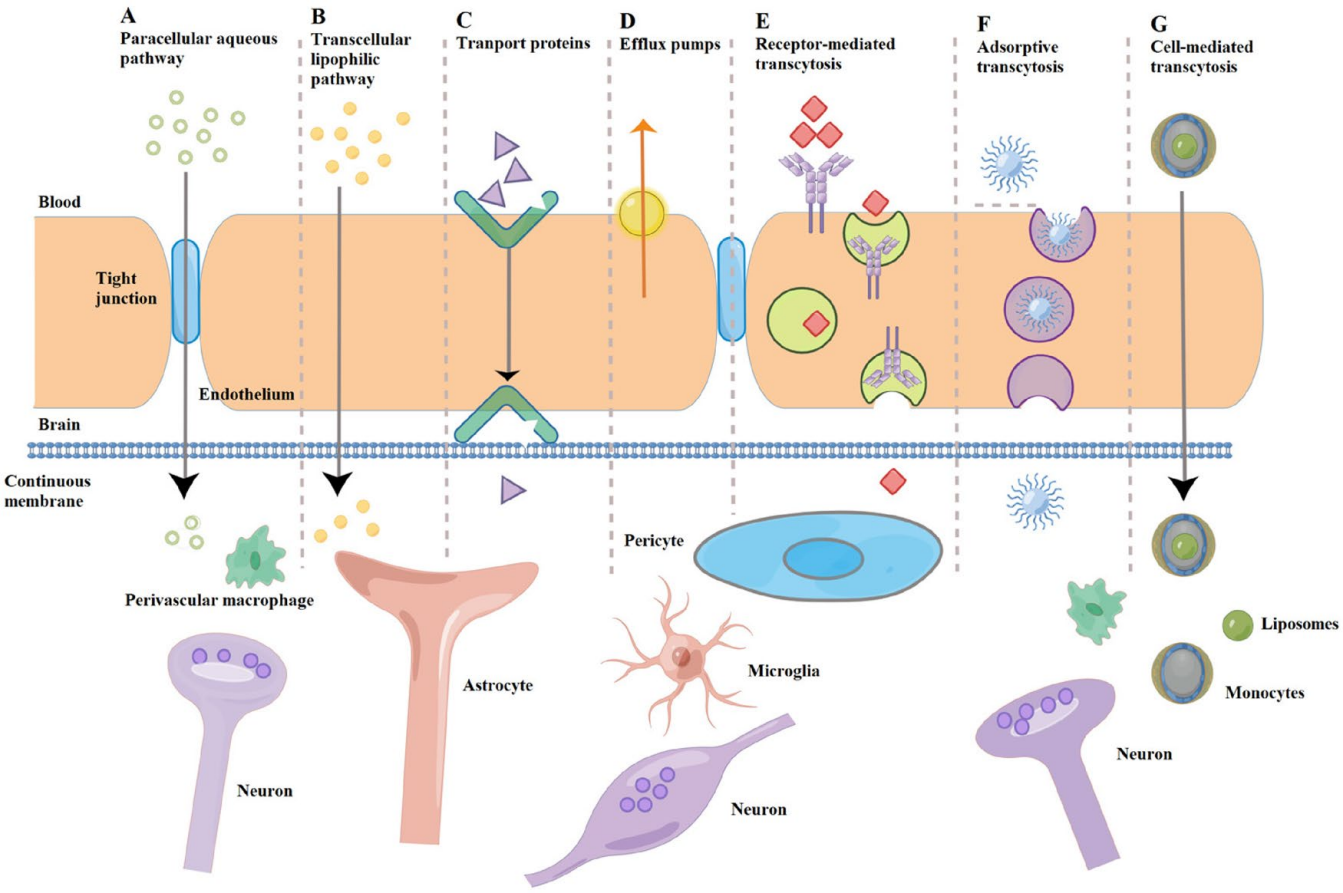
Schematic diagram of drug transport across the BBB (Drawn by Figdraw).

Paracellular and transcellular transport are the main routes for substances to cross BBB. The paracellular transport is restricted by the tight junction between endothelial cells, and can only transport micromolecule (such as CO_2_, O_2_, H_2_O and C_2_H_5_OH) across the BBB. Transcellular transport includes passive diffusion and endocytosis (Hersh et al., 2016), and the former is only applicable for lipophilic drugs below 500 Da (Grabrucker et al., 2016). Therefore, the transport of drugs across BBB mainly depends on endocytosis, including carrier-mediated, receptor-mediated, adsorption-mediated and cell-mediated transport (Figure 2) (Chen & Liu, 2012).

#### 3.2.1. Carrier-mediated transport

**Figure 3.**
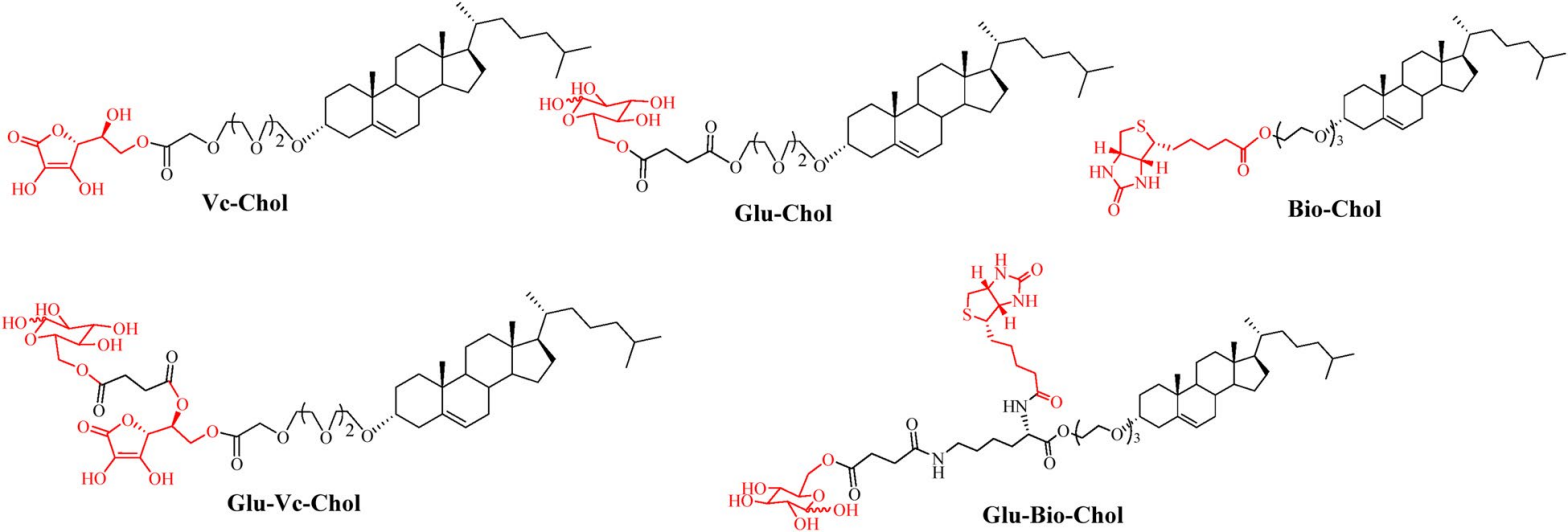
The structures of liposome ligands Glu-Chol, Vc-Chol, Bio-Chol, Glu-Vc-Chol, and Glu-Bio-Chol.

There are many transporters on the BBB for the transportation of nutrients, such as the glucose transporter 1 (GLUT1), vitamin C transporter 2 (SVCT2), Na^+^-dependent vitamin transporter (SMVT), L-amino acid transporter 1 (LAT1), monocarboxylic acid transporter 1 (MCT1), et al (Zhao et al., 2014; Jiang et al., 2021; Zhao et al., 2021). The high affinity between these transporters and substrates facilitates the substrate crossing the BBB through carrier-mediated transport. Modification of the NDDS with the substrates or their analogues can promote drugs entry into the brain. As shown in Figure 3, our group has designed the liposome ligands modified with glucose, vitamin C, biotin, glucose-vitamin C (Glu-Vc), and glucose-biotin (Glu-Bio) to enhance the drug transport through the highly expressed GLUT1, SVCT2, SMVT transporter on the BBB, which significantly improves the ability of drugs to enter the CNS in different degrees (Lei et al., 2011; Peng et al., 2018; Huang et al., 2020).

#### 3.2.2. Receptor-mediated transport

**Figure 4.**
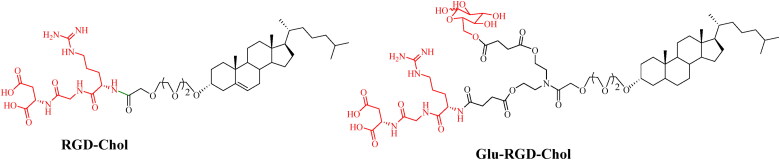
The structures of liposome ligands RGD-Chol and Glu-RGD-Chol.

Receptor-mediated transport is the primary way for internalizing large biomolecules and growth factors in the brain, and is the most widely used strategy in brain tumor targeted drug delivery. The low density lipoprotein receptor (LDL-R), apolipoprotein E (ApoE) receptor, epidermal growth factor receptor (EGFR), transferrin receptor (TfR), insulin receptor (IR) and integrin receptor (αvβ3) are the commonly used targets that promote the delivery of drugs into the brain. Therefore, the ligands modification on the NDDS can specifically bind to the receptor on BBB, which effectively increases the drug concentration in the brain. In our previous studies, the RGD-modified liposomes that could be mediated by αvβ3 across the BBB (Figure 4), and the distributions of drugs in brain are improved with 2.44 and 4.72 times than that of naked drug (Fu et al., 2019).

#### 3.2.3. Adsorption-mediated transport

When the nanoparticles are modified with cationic components, such as protamine, cell-penetrating peptide (CPP), etc., they bind to the anion membrane of brain microvascular endothelial cells to promote endocytosis by the cells. However, the adsorption-mediated transport does not involve the specific binding to the cell membrane, so the cationic nanoparticles can also enter normal tissues and cause inevitable side effects (Meng et al., 2017). The CPPs are the most frequently-used ligands for adsorption-mediated transport, which remain electropositive under physiological conditions due to the abundant arginine and lysine residues. What’s more, the interaction with anionic substances on the endothelial cell membrane further enhances the cell uptake (Herve et al., 2008). Due to the lack of specific adsorption of CPP on tumor cells, most studies utilize the CPP combined with tumor targeted ligands to prepare the dual- or multiple-targeting NDDS. For example, Sun et al. synthesize the copolymer TfR-T12-PEG-PLGA targeting transferrin receptor and the CPP-modified polymer TATH7-PEG-PLGA (Sun et al., 2022). The nano polymer micelles are prepared with the polymers for synchronously delivery of paclitaxel (PTX) and immunomodulator imiquimod. The modification of TfR-T12 peptide can achieve the targeted delivery of chemotherapeutic drugs through the BBB mediated by TfR. The pH-sensitive TATH7 can increase the uptake efficiency for the micelles by tumor cells through adsorption-mediated in pH 5.5 medium than that under pH 7.4 medium. Therefore, the micelles have enhanced the therapeutic effect on brain tumors through chemotherapy and immunotherapy.

#### 3.2.4. Cell-mediated transport

Nanoparticles can also cross the BBB through cell-mediated transport, called ‘Trojan’. In general, leukocytes and stem cells are widely used as carriers for cell-mediated transport to deliver nanoparticles to the target region. These biomimetic delivery systems have unique advantages compared with other delivery systems, including prolonged blood circulation time and biological half-life, low immunogenicity, and enhanced biocompatibility (Charabati et al., 2020). In recent years, the application of several leukocytes, such as neutrophils and mononuclear macrophages, has made good progress in brain-targeted drug delivery across the BBB (Pang et al., 2018; Wu et al., 2018). Wu et al. developed the inflammation-activatable engineered neutrophils by internalizing doxorubicin-loaded magnetic mesoporous silica nanoparticles (ND-MMSNs) (Wu et al., 2018). After systemic injection of ND-MMSNs, the nanoparticles migrate along the molecular guidance signals, and accumulate in the inflamed glioma sites. Subsequently, highly activated neutrophils carrying D-MMSNs release neutrophil extracellular traps in the inflammatory region. In the meanwhile the drug-loaded nanoparticles were released and uptaken by infiltrating glioma cells, achieving visualization of diagnosis and treatment of postoperative glioma. Pang et al. prepared M1 macrophage-loaded nanoparticles (M1-NPs) by incubating poly(lactide-co-glycolide) nanoparticles with primary M1 macrophages for glioma therapy (Pang et al., 2018). The macrophages that present a strong phagocytic capacity to incorporate the drug-loaded NPs were able to effectively migrate and infiltrate into orthotopic glioma tumor models for DOX release. It was noteworthy that M1-NPs significantly prolonged mice survival with median survival 38.5 days (PBS group, 21 days). What’s more, the M1-NPs also increased caspase-3 protein expression. All the results indicated that DOX@M1-NPs exhibited a significant improvement in anti-tumor activities.

#### 3.2.5. Reducing drug efflux

As mentioned above, the high expressed efflux proteins (such as P-glycoprotein, P-gp) on the BBB are also important factors that affect the entry of drugs into brain tissue. Therefore, reducing the expression of efflux proteins or inhibiting its activity is an important means to increase the drug concentration in the CNS to improve the therapeutic effect and reverse drug resistance. For example, the ‘cocktail’ liposomes co-loading with verapamil and riluzole overcome drug resistance by inhibiting P-gp in brain endothelial cells and astrocytes (Tang et al., 2015).

#### 3.2.6. Overcoming the blood-brain tumor barrier

Compared with BBB, BBTB has the higher permeability, so the nanoparticles can cross BBTB through the EPR effect. On the other hand, many receptors are overexpressed on BBTB, such as EGFR, matrix metalloproteinase-2 (MMP-2), TfR, interleukin-13 receptor (IL-13R), etc, are widely used for targeting BBTB. The ideal brain targeted drug delivery systems could not only overcome the barriers of BBB, but also overcome BBTB and selectively target cancer cells, thereby reducing the distribution in normal brain cells. Therefore, it is urgently needed to develop a dual-targeted drug delivery system with BBB-targeting and BBTB-targeting capabilities. The widely used carriers/receptors are highly expressed in the cells and their ligands are shown in Table 1. Our group have designed a novel dual-targeting ligand modified with glucose and RGD (Glu-RGD-Chol, Figure 4). PTX-loaded liposome is prepared with this ligand, which contributes to crossing BBB and targeting glioma (Fu et al., 2019). Compared with naked PTX, the brain targeting performance of this dual targeting liposome was increased with 4.41 times.

**Table 1.** The transporters/receptors and their ligands targeting blood-brain barrier and brain tumors.

	Target	BBB	Tumor	Ligand
Transporter	GLUT	√	√	Glucose, Glucose analogues (2-deoxy-D-glucose), other hexoses (Mannose, Galactose, Glucosamine)
	GSH transporter	√	×	GSH
	LAT1	√	×	Large neutral amino acids (L-tyrosine, L-phenylalanine, L-leucin, L-isoleucine)
	MCT_1_	√	×	Lactate, Pyruvate, Biotin, Sialic acid
	Nucleoside transporter	√	×	Adenosine, Guanosine, Uridine
	Choline transporter	√	×	Choline, Thiamin
Receptor	TfR	√	√	Tf, OX26, T7, TfR-lytic hybrid peptide
	LR	√	√	Lf
	LDLR	√	√	nLDL, Peptide-22
	LRP	√	√	Angiopep-2, MTf, ApoE, Peptide-22
	Insulin Receptor	√	√	83-14 murine monoclonal antibody
	Integrin αvβ3	√	√	RGD, c(RGDyK), cRGD
	EGFR	√	√	GE11, EGF, mAb225
	TGN receptor	√	×	TGN peptide
	CD44	×	√	HA
	IL-13 Receptor	×	√	IL-13
	FR	×	√	Folate, Pteroic acid

### 3.3. Stimulate-responsive nano drug delivery system

**Figure 5.**
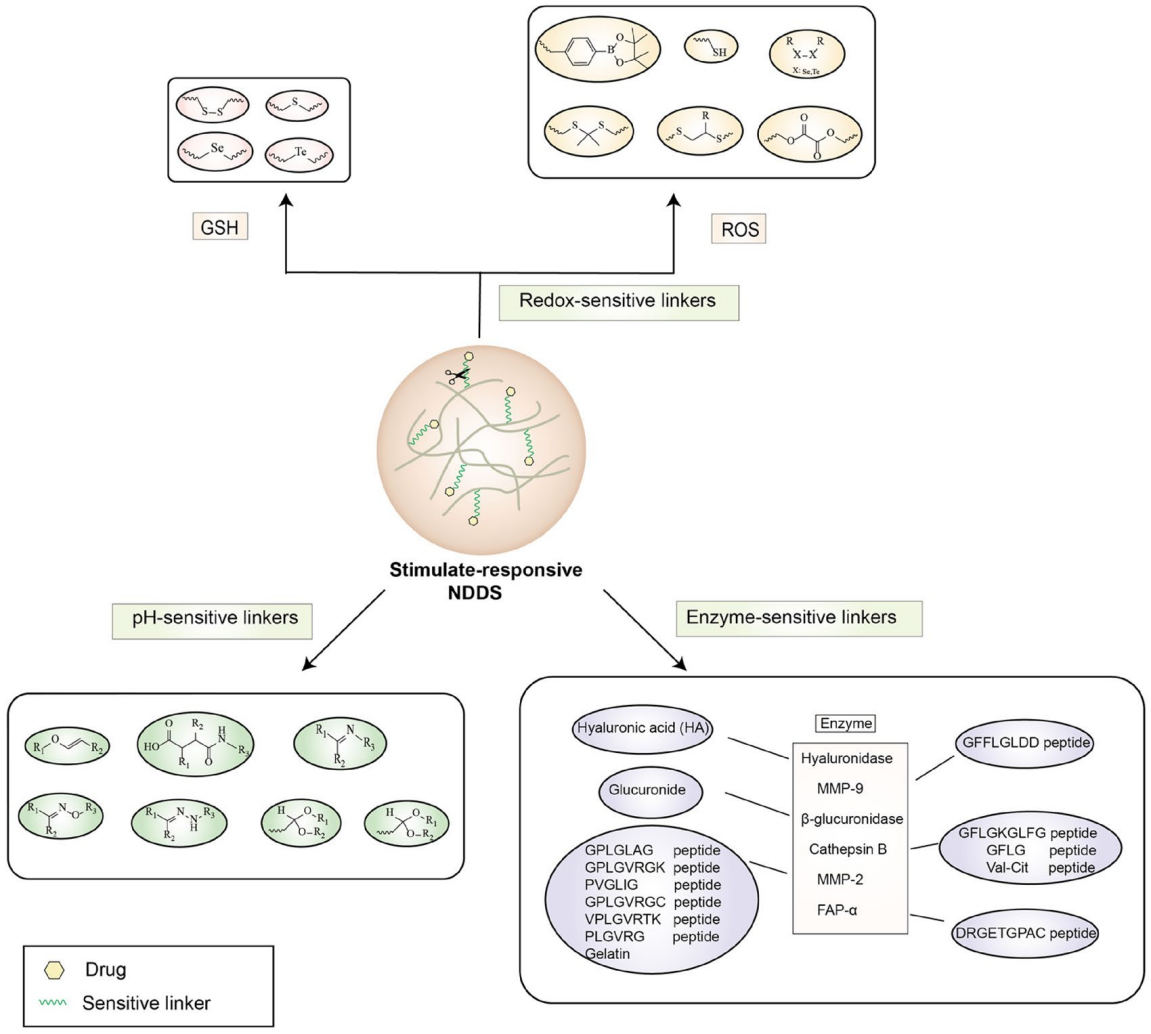
Schematic diagram of stimulate-responsive nano drug delivery system.

The active-targeting strategy depends on the high expression of specific receptors/transporters in tumors, and the heterogeneous expression may greatly reduce its targeting efficiency (Srinivasarao et al., 2015). In addition, most receptors/transporters are also expressed in normal tissues, potentially causing off-target effect. Therefore, the active-targeting strategy based on ligand modification is not enough to achieve efficient delivery of drugs for brain tumors. The ‘stimulate-responsive’ strategy is emerged for drug delivery by introducing sensitive groups. The sensitive groups will undergo physical and chemical changes (such as protonation, deprotonation, fracture or degradation) when the nanoparticles are stimulated at the pathological site, resulting in structural changes in the nanoparticles (Figure 5). These changes help to enhance cellular uptake, release drugs, promote lysosomal escape, turn on imaging signals, and penetrate into tumors (Chen et al., 2017). Therefore, the ‘stimulus-response’ strategy can improve the bioavailability of antitumor drugs and the antitumor efficacy.

#### 3.3.1. Ph-responsive nano drug delivery system

The decreased pH in extracellular and interstitial is a sign of malignancy, which is due to the excess of metabolites (lactic acid, CO_2_, etc), as well as the increased expression and activity of vacuolar (V-type) H^+^-ATPase (Helmlinger et al., 2002). Compared with normal tissue (pH 7.0 ∼ 7.4), the extracellular pH of the tumor can be reduced to 5.6 ∼ 6.8. In addition, the pH in lysosomes is only about 4.5 ∼ 5.5 (Shi et al., 2020).

Generally, pH-sensitive materials work based on the following two mechanisms: the cleavage of acid sensitive chemical bonds and the protonation of materials. Various chemical functional groups, such as (semi) acetals, amides, orthoesters, amines, imines, and hydrazones, can be used as pH-sensitive groups are showed in Table 2 (Shi et al., 2020). The structures of commonly used acid sensitive chemical bonds and their degradation products (Shi et al., 2020). He et al. prepared the pH-sensitive polymer vesicles (Au-DOX@PO-ANG) to deliver the gold nanoparticles-doxorubicin complex to treat glioma (He et al., 2021). The tertiary amide group binds to hydrogen ions in solution and forms hydrogen bonds under acidic conditions, which would disrupt the core-shell structure, thereby reducing the stability of the polymersomes and releasing the drugs.

**Table 2.** pH-sensitive chemical bonds and degradation products.

Type	Acid sensitive chemical bonds	Degradation products
Vinyl ester	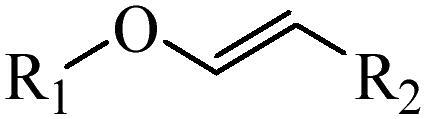	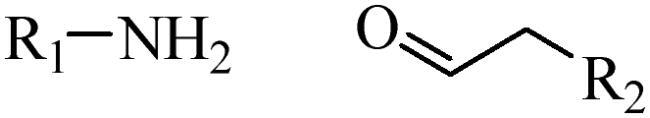
Amide	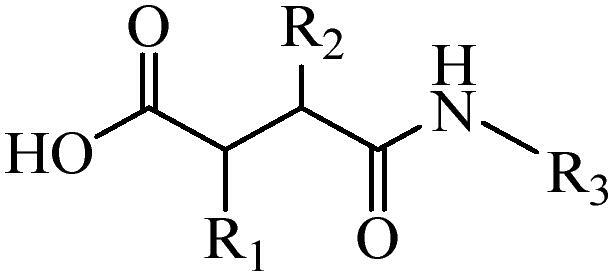	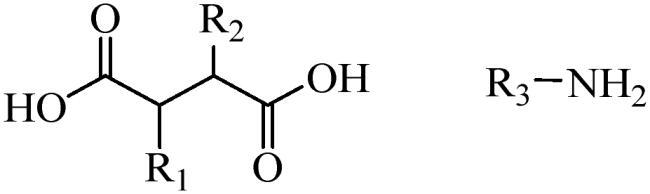
Imine	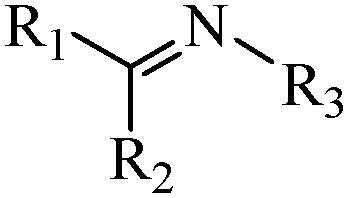	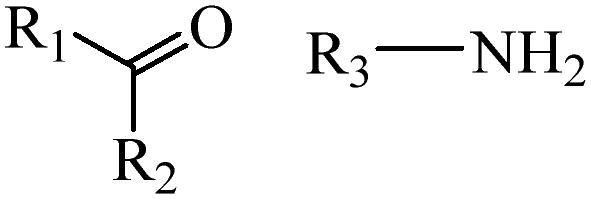
Oxime	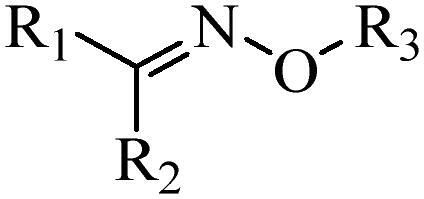	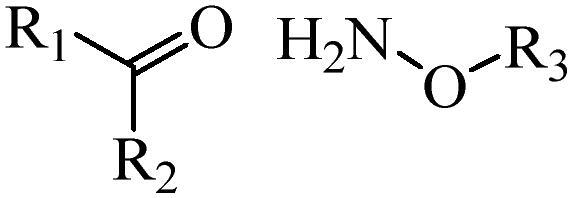
Hydrazone	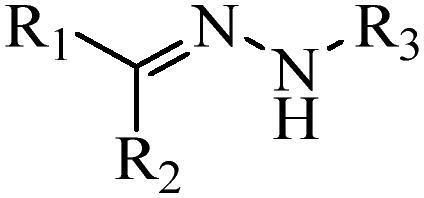	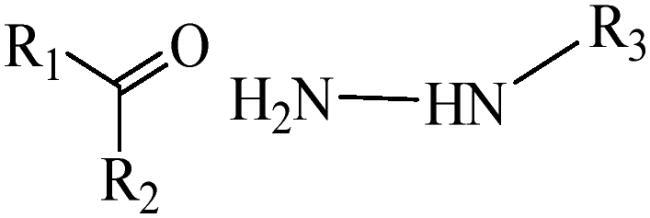
Acetals	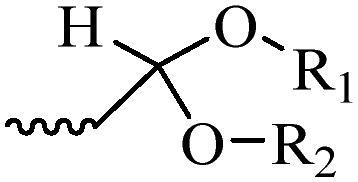	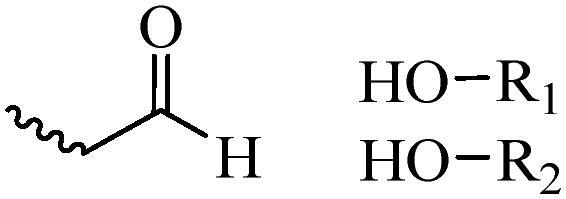
Orthoester	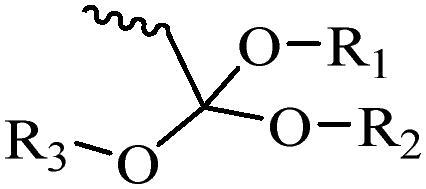	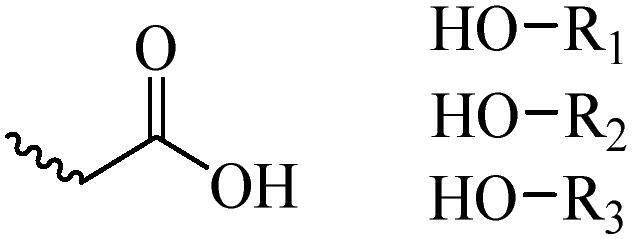

#### 3.3.2. Redox-responsive nano drug delivery system

Compared with healthy tissues, the tumor tissue is highly reduced. As the main contributor to the redox state in cells, the concentration of glutathione (γ-Glutamyl-cyste-glycine, GSH) is about 0.5 ∼ 10 mm, which is more than 100 times higher than that in healthy cells (2 ∼ 20 µm) (Zhao et al., 2021). Disulfide bond, sulfide, selenide and telluride are commonly used functional groups for designing GSH-sensitive nano carriers. The structures and their products after reacting with GSH are shown in Table 3. Wen et al. prepare angiopep-2 (AP)-modified redox-responsive nanoparticles to co-deliver siVEGF and PTX for glioma targeted therapy (Wen et al., 2020). The disulfide bond was broken by GSH, which allows the cleavage of the nano carrier and release of the drugs. As anticipated, Ap-CSssSA/P/R showed slower release during the whole experiment period with 0 mM GSH, however, PTX and siVEGF releases were significantly enhanced with 10 mM GSH, and the cumulative release was over 90% after 48 h.

**Table 3.** The widely used GSH- and ROS-sensitive chemical bonds and broken products.

	Type	Chemical bonds	Degradation products
GSH	Disulfide	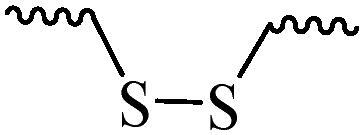	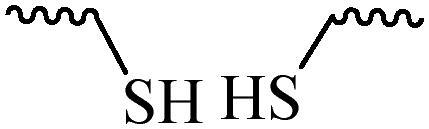
	Thioether	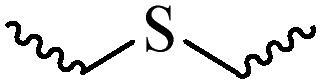	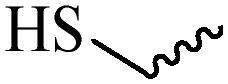
	Selenium	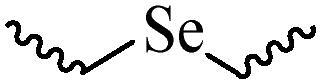	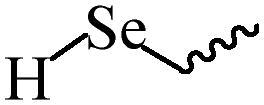
	Telluride	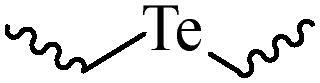	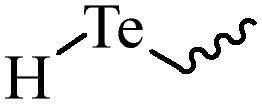
ROS	Arylboronic esters	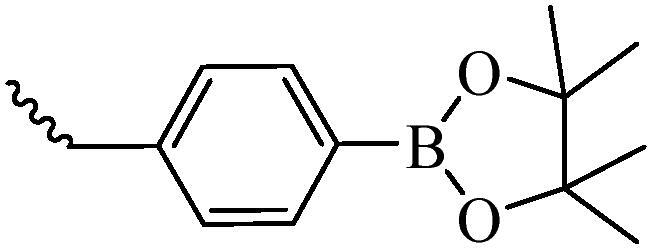	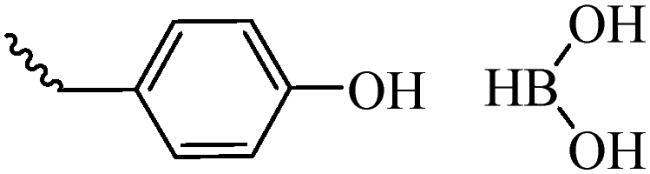
	Sulfhydryl	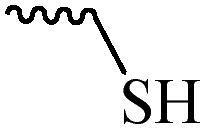	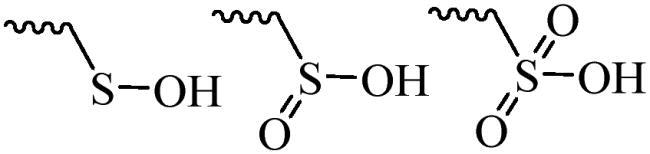
	Diselenide/Ditelluride	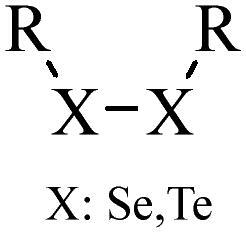	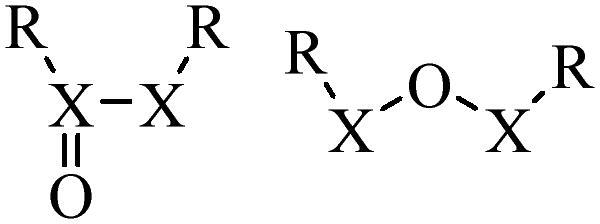
	Thioketal	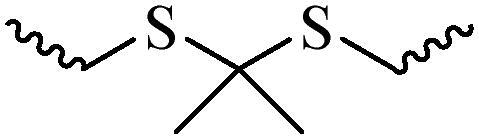	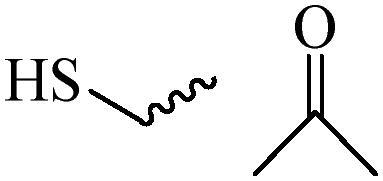
	Thioether	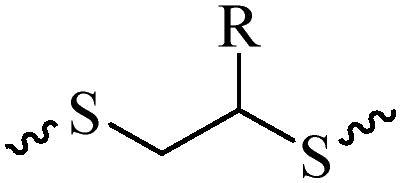	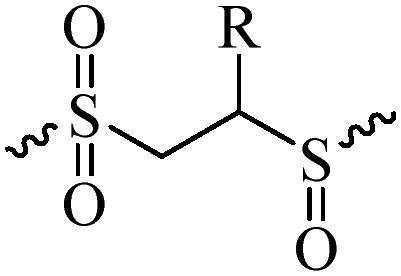
	Peroxalate ester	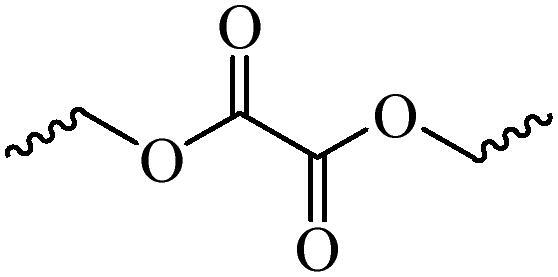	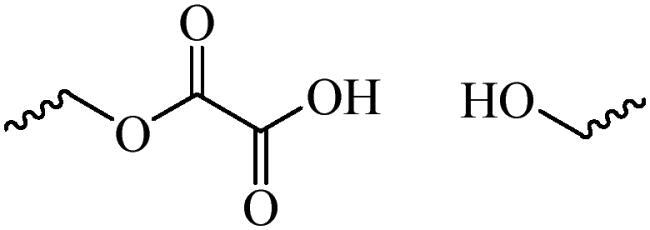

Rapid cell proliferation and high metabolic rate lead to a higher level of ROS (100 µm) in the tumor environment, much higher than that in the normal tissues (20 nm) (Xu et al., 2017). Table 3 lists the structure of commonly used ROS-responsive groups and their products after reacting with ROS. Zheng et al. design an ROS-responsive siRNA nanomedicine, 3I-NM@siRNA stabilized by ‘triple interactions’ (Zheng et al., 2019). 3I-NM@siRNA exhibited an active ROS response and efficient siRNA release upon treatment with H_2_O_2_, whereas 2I-NM@siRNA was very stable and no siRNAs were released. When 3I-NM@siRNA encounters ROS stimulus inside brain tumor, the hydrophobic phenylboronic ester is converted to its hydrophilic counterpart with carboxyl groups, which depletes the hydrophobic stabilization force and subsequently the newly produced carboxyl groups interfere with electrostatic and hydrogen bond interactions. This sequential ‘self-destruct’ process enables effective siRNA release.

#### 3.3.3. Enzyme-responsive nano drug delivery system

The abnormal expression of enzymes has been used as a agonist for drug delivery and tumor targeting (Park et al., 2021). Some commonly used enzymes and their substrates are shown in Table 4 (Park et al., 2021), among which, MMPs, cathepsin B, hyaluronidase (HAase) and β-glucuronidase are widely used in NDDS. MMPs, members of the proteolytic enzyme family, are overexpressed in many types of tumors and play a key role in degrading extracellular matrix and promoting tumor metastasis (Shahriari et al., 2019). Hua et al. design and develop a dual-functional peptide-drug conjugate, SynB3-PVGLIG-PTX (Hua et al., 2021). The PTX binds to SynB3 through an MMP-2-sensitive linker (PVGLIG), which helps drug release at the target sites with high MMP-2 expression level. In the presence of MMP-2, SynB3-PVGLIG-PTX could completely disappear, while the percentage of PVG-PTX peaked (100%). In contrast, SynB3-PVGLIG-PTX could not be cleaved without MMP-2, and neither PVG-PTX nor free PTX could be detected.

**Table 4.** The commonly used enzymes and their substrates.

Enzyme	Linker
Hyaluronidase	Hyaluronic acid (HA)
MMP-9	GFFLGLDD peptide
MMP-2	GPLGLAG peptide, GPLGVRGK peptide, PVGLIG peptide, GPLGVRGC peptide, VPLGVRTK peptide, PLGVRG peptide, Gelatin
Cathepsin B	GFLGKGLFG peptide, GFLG peptide , Val-Cit peptide,
FAP-α	DRGETGPAC peptide
β-glucuronidase	Glucuronide

### 3.4. Organelle targeting

**Figure 6.**
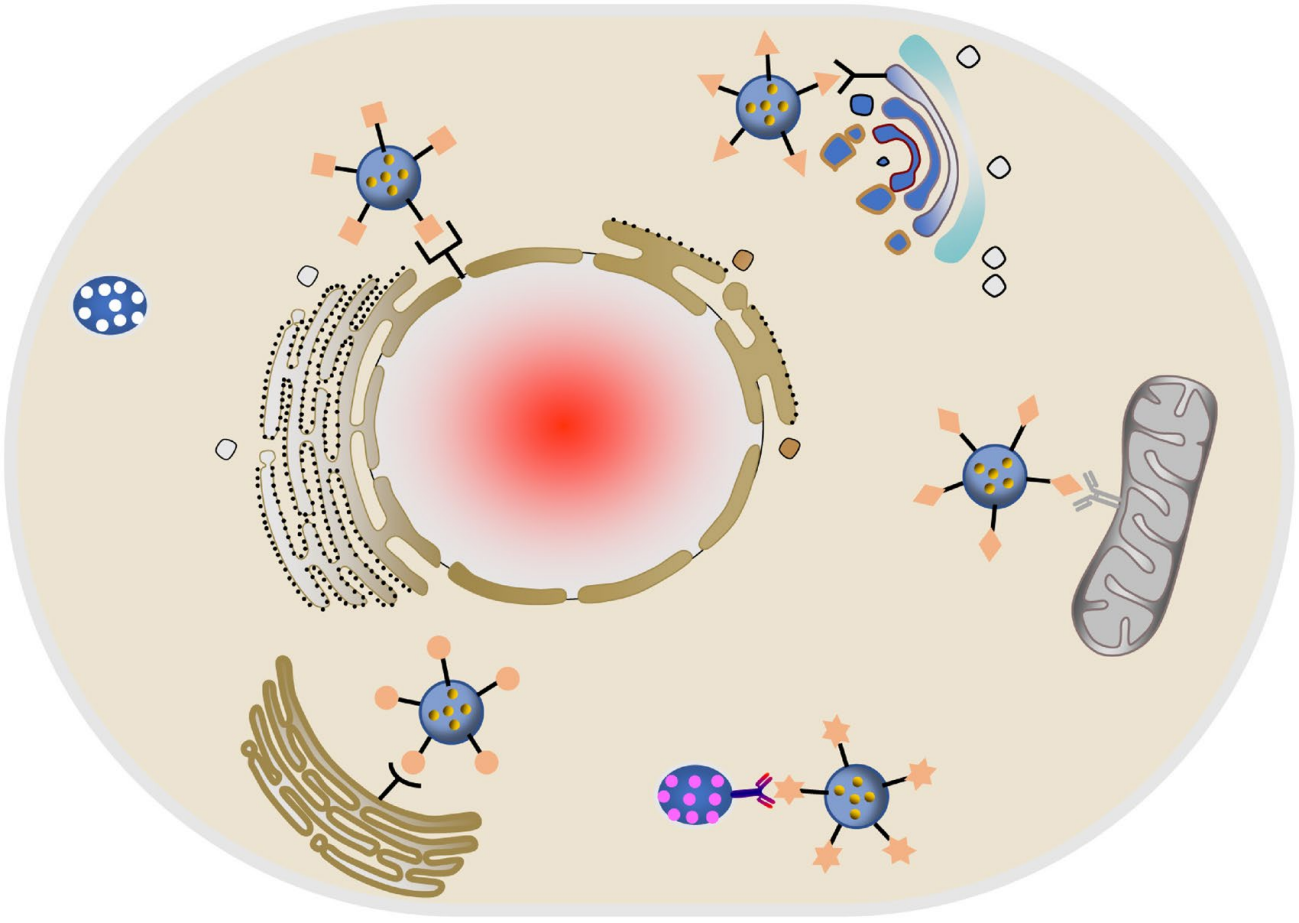
Schematic diagram of organelle targeting nano drug delivery system.

In recent years, the nano materials with subcellular-targeting abilities have attracted much attention in the field of cancer therapy (Jin et al., 2020). Based on the molecular mechanism of the drug, targeting subcellular organelles has gradually become an important part of precision medicine (Gu et al., 2021). For example, drugs that produce reactive oxygen species are delivered to mitochondria, and therapeutic agents that blind DNA are delivered to the nucleus (Vankayala et al., 2015; Chen et al., 2019). Transporting drugs into the target organelles can maximize the efficacy of drugs, which is conducive to completely eradicating tumors and preventing tumor recurrence, invasion and metastasis (Figure 6) (Chen et al., 2019).

#### 3.4.1. Nucleus targeting

Many anti-tumor drugs widely used in the clinic are toxins related to DNA replication, and the drugs inhibit DNA replication by interacting with DNA or inactivating related enzymes. Therefore, delivering these drugs into the nucleus contributes to improving the efficacy (Vankayala et al., 2015). At present, the design of nuclear targeted nano-carriers can be summarized into the following two methods: (1) modifying nano-carriers with nuclear-targeted peptides to promote the enrichment of drugs in the nucleus; (2) preparing the nano-carriers with switchable size (Wei et al., 2021).

**Figure 7.**
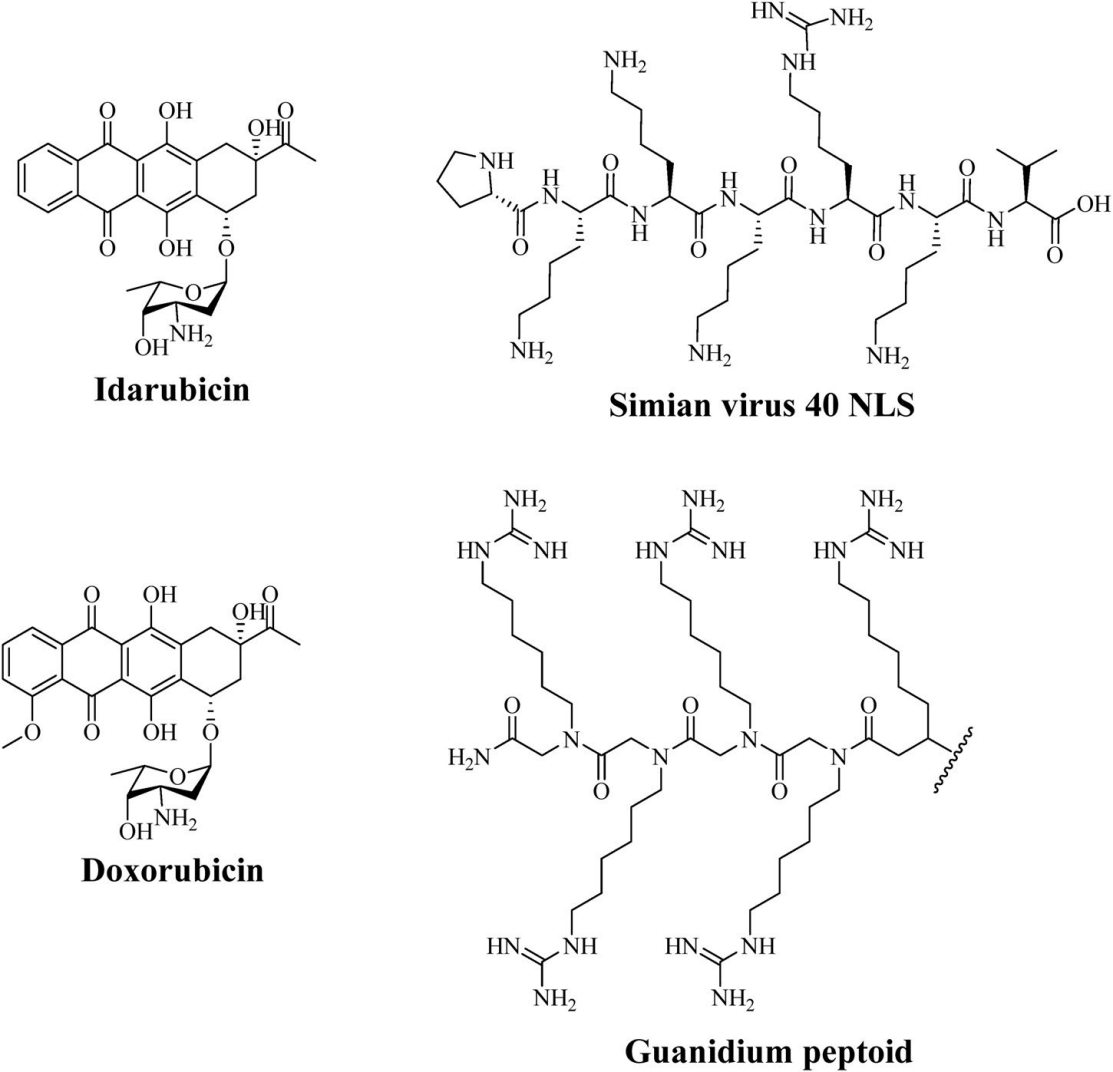
The structure of several commonly used nucleus targeting groups.

The first method is to modify the nano-carriers with specific ligands that can activate and internalize nuclear receptors to promote the interaction between carriers and nuclear membranes, so as to promote the entry of nanoparticles into the nucleus (Tanaka et al., 2017). The nuclear localization sequence (NLS) is a short peptide that is rich in lysine, arginine or proline, which can transfer the molecules attached to the nucleus through the nuclear pore. The structures of several commonly used nuclear targeting groups are shown in Figure 7 (Lange et al., 2010). For example, CB5005, a nuclear targeting peptide, is composed of membrane permeation sequence (CB5005M) and nuclear localization sequence (CB5005N), which can significantly enhance the enrichment of drugs in the cytoplasm and nucleus. In addition, CB5005 can also target intracellular NF-κB and inhibit its activation. Co-administration of CB5005 and doxorubicin (DOX) shows a synergistic effect in anti-glioma (Louzoun-Zada et al., 2019).

Nuclear pores control the transport process between cytoplasm and nucleoplasm. The diameter of the pores is only about 39 nm, which makes it difficult for nanoparticles to penetrate the nucleoplasm through passive diffusion (Haddad et al., 2020). To enhance the penetration capacity of nano drugs into nucleus, Wang et al. develop a pH- and GSH-responsive micelle. The charge reverses in the tumor microenvironment, so as to contribute to the entry into tumor cells through an adsorption-mediated effect. In the presence of GSH, the disulfide bond is broken and the particle size becomes smaller. In addition, surface conjugated dexamethasone can effectively dilate the nuclear pores, which facilitates the free entry of micelles into the cell nucleus (Wang et al., 2017).

#### 3.4.2. Mitochondria targeting

As the ‘power room’ of eukaryotic cells, mitochondria are responsible for energy production, electron transmission, calcium metabolism, ROS production and immune regulation (Cho et al., 2020). Therefore, the function changes will affect biosynthetic pathways, cell signal transduction, chromatin structure and the activation of apoptosis. Targeted delivery of drugs to mitochondria and regulation of mitochondrial function provide great potential for the treatment of tumors.

**Figure 8.**
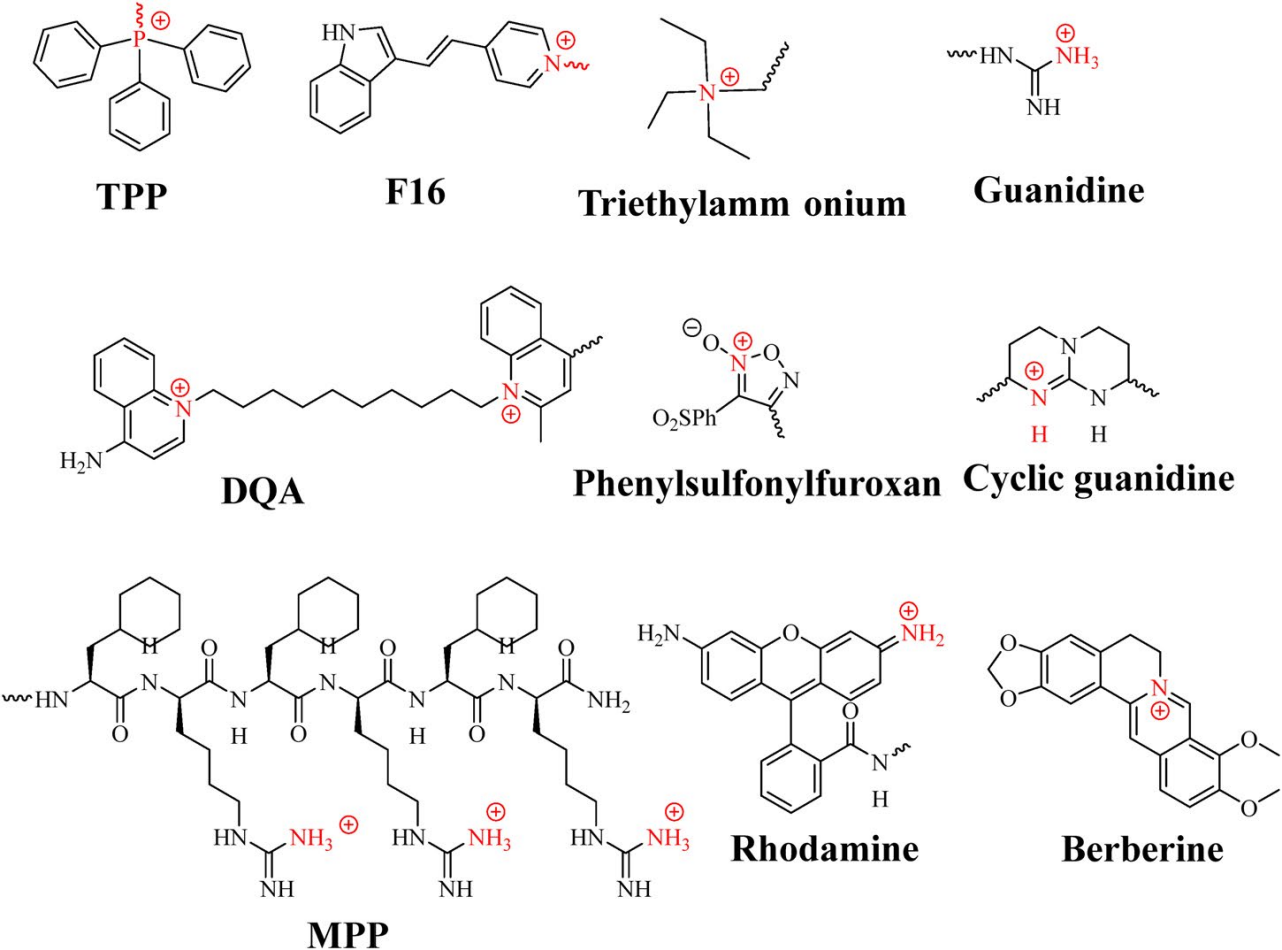
The structure of molecules targeting mitochondria.

The mitochondrial membrane potential (-160 ∼ −180 MV) of malignant cells is higher than that of normal cells, which indicates that it is feasible to selectively target the mitochondria of tumor cells. In order to enter the mitochondria, compounds must pass through the cell membrane and mitochondrial membrane. Fortunately, the both membrane potentials are negative, which allows the cationic compounds to accumulate initially in the cell cytosol and then inside the mitochondria. At present, several molecules targeting mitochondria have been reported (Figure 8), such as mitochondrial penetrating polypeptide (MPP), delocalized lipophilic cations such as triphenylphosphonium (TPP), rhodamine, berberine, guanidine and (E-4-(2-(indole-3-yl) vinyl)-1-methylpyridinium salt (F16), etc (Louzoun-Zada et al., 2019).

#### 3.4.3. Lysosome targeting

Lysosome, the digestive organs in cells, containing many hydrolases for degrading, repairing and recycling biomolecules, which plays a crucial role in autophagy, secretion, and membrane repair (Yu et al., 2010). Nanomaterials are attractive in lysosome targeting because most nano preparations are eventually transported into lysosome via membrane receptor-mediated endocytosis. Therefore, there are few studies on promoting the entry of nanoparticles into the lysosome through ligand modification. On the contrary, there are many studies on lysosome escape, for example, cationic liposomes are more likely to escape from the lysosome through the ‘proton sponge’ effect. While, there are still some nano preparations that functionalized by lysosome-targeting groups to improve the accumulation in lysosome and further improve anticancer efficiency (Wang et al., 2016). For instance, mannose 6-phosphate is a promising lysosomal guiding group that can promote the entry into lysosome (Coutinho et al., 2012). Delivery vehicles using M6P-decorated nanoparticles have been developed for anti-cancer therapy. Crucianelli et al. developed M6P-decorated liposomes made of a M6P cholesteryl conjugate where a sufficiently rigid aryl-incorporated linker connects the M6P moiety to a steroid structure and ensured exposure of the M6P function to favor tight association with the liposome with the receptor (Crucianelli et al., 2014). And the enhanced uptake of M6P-decorated liposomes in cancer cells was confirmed. Furthermore, this group prepared another M6P-decorated liposomes loading with C6-ceramide, which behaves as a detergent and was found to induce lysosomal membrane permeabilization, in order to target lysosomes in cancer cells and induce apoptosis (Minnelli et al., 2018).

#### 3.4.4. Endoplasmic reticulum targeting

Endoplasmic reticulum (ER) plays an important role in protein synthesis, folding and post-translational modification. In addition, ER is also involved in lipid biosynthesis, maintaining calcium homeostasis and other physiological functions. At present, the commonly used strategy for ER-targeting is to modify the nanoparticle with an ER-targeting peptide, such as the KKXX and KXKXX search signal, RXR retention/recovery signal, and the KDEL (Lys-Asp-Glu-Leu) retention/recovery signal (Ma et al., 2016). In addition, some small molecular groups such as p-toluenesulfonamide, hydrazide can also be used to target ER. Wang et al. design gold nanoparticles (KDEL-AuNPs) modified with KDEL, which can be internalized and accumulated in ER (Wang et al., 2013).

#### 3.4.5. Golgi targeting

Golgi is the site for post-translational protein modification, and the structural integrity of golgi is important for certain signaling pathways, especially those related to migration, invasion and angiogenesis (Wang et al., 2015; Nishita et al., 2017; Yu et al., 2018). Therefore, the destruction of golgi structure in tumor cells may be a potential method to destroy multiple signaling pathways and a good strategy for targeted tumor therapy. Gong’s group has developed a golgi-targeted prodrug nanoparticles by combining chondroitin sulfate (CS) with retinoic acid (RA). This nanoparticles accumulate in the golgi of cancer cells and then release RA in the acidic environment. The evaluation in vitro and in vivo further confirms that CS-RA inhibits the expression of many metastasis-related proteins by destroying the golgi structure. After loading with PTX, the CS-RA based nanoformulation (PTX-CS-RA) suppresses tumor growth and metastasis (Li et al., 2019).

## 4. Drug combination strategy based on multifunctional nano drug delivery system

In recent years, combination therapy has received attention for decreasing side effects and increasing efficacy (Zhao et al., 2020). At present, the combination therapy in clinical research for brain tumors includes the combination of chemotherapy drugs (temozolomide, paclitaxel, camptothecin, methotrexate, etc.), chemotherapy drugs and small molecule targeted drugs (sildenab + lomustine, bortezomib + temozolomide), t small molecule targeted drugs (erlotinib + vorinolta), small molecule drugs and monoclonal antibodies (bevacizumab + irinotecan), small molecule drugs and nucleic acid drugs (temozolomide + SGT-53), etc (Zhao et al., 2020). Although these combination therapies have improved the clinical outcomes to some extent, they are still not ideal due to the complexity of brain tumors. Therefore, there is an urgent need to improve the existing combination strategies to solve these problems, and the combined drug therapy based on multi-functional drug delivery system has made good progress in brain tumors (Khan et al., 2021).

**Figure 9.**
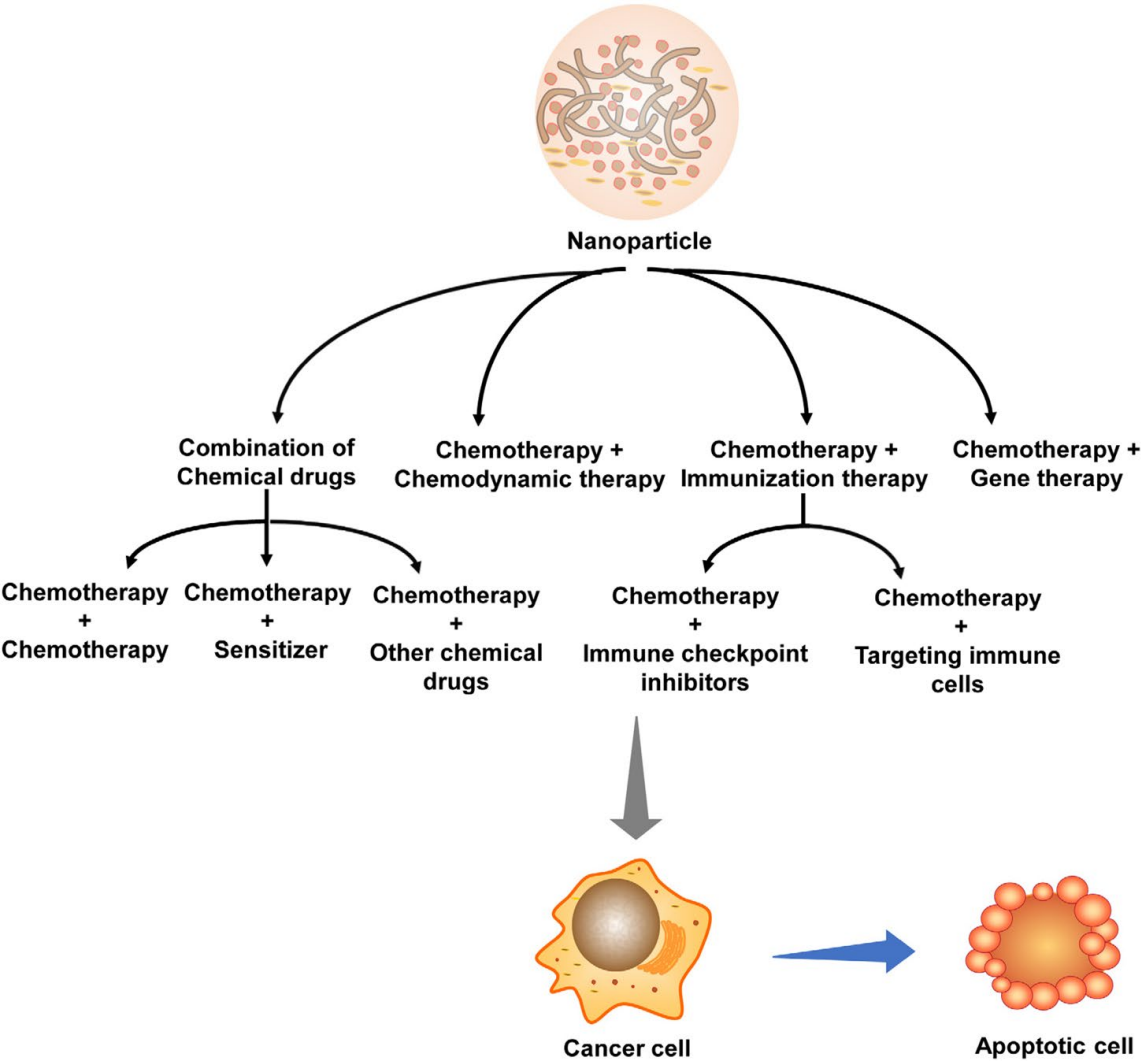
Combination strategy for brain tumors.

Two or more drugs with different anti-tumor mechanisms are commonly selected for combined administration, such as chemotherapy drugs with different mechanisms, chemotherapy drugs + chemotherapy sensitizers, chemotherapy drugs + small molecule targeted drugs, and small molecule drugs and biological drugs (chemotherapy drugs + monoclonal antibodies, chemotherapy drugs + immune agents, chemotherapy drugs + nucleic acid drugs), et al (Figure 9). The combination of drugs with different mechanisms has the following advantages. Firstly, the drugs kill tumor cells against different targets to improve the efficacy. Secondly, the combined administration can reduce the dose, thereby decreasing the toxic and side effects. It can also avoid the drug resistance caused by a single drug. In addition, the administration can improve the killing effect on tumor stem cells and metastatic tumor cells, so as to reduce the recurrence of the disease. What’s more, the proportion and the release sequence of drugs are also important for the synergistic treatment. This section will summarize the application of multifunctional NDDS in the combined treatment of brain tumors, including the combined use of small molecular chemotherapy drugs, chemotherapy-immunotherapy, and chemotherapy-gene therapy.

### 4.1. The combination of small molecular chemical drugs

#### 4.1.1. The combination of chemotherapy drugs

The chemotherapy drugs have a limit in specificity targeting the tissue, which may lead to the obvious toxic and side effects, and MDR. Therefore, the combination of two or more chemotherapeutic drugs with different mechanisms is the most commonly used strategy (Shrestha et al., 2021).

Zhang et al. designed a dual-targeting ligand modified with lactoferrin (Lf) and RGD peptide to prepare lipid nanoparticles (L/R-T/V-NLC) loading with TMZ and vincristine (VCR). The RGD peptide can recognize the α_v_β_3_ receptor overexpressed on neurovascular endothelial cells and the Lf can recognize transferrin receptor (TFR) on the brain tumors to facilitate the nanoparticles crossing the BBB and then targeting tumor cells. The results show that L/R-T/V-NLCs inhibited the tumor growth better than that treated with single-ligand-modified NLCs, single-drug-loaded NLCs, and drug solutions (Zhang et al., 2018). Xu et al. prepare the nanoparticles with the cationic micellar core loading curcumin and the anion shell loading DOX to target both cancer cells and stem cells (Xu et al., 2018). And an obvious inhibition of tumor growth was observed after treatment with the nanoparticles. The rat survival after treatment with this nanoparticles (64.5 days) was significantly prolonged in comparison to the control group (31.4 days), the combinational Cur/DOX solution group (36.3 days), the DOX-VPDP group (33.5 days) and the Cur-VPDP group (38.7 days). Liu et al. develop the HA-grafted micelles encapsulating lauroyl-gemcitabine and honokiol. The micelles penetrate into the tumor sphere mediated by the CD44 receptor, and enhances the cytotoxicity to glioma cells (Liu et al., 2018). In vivo, drug-loaded HA-M significantly increased the survival rate of mice bearing orthotopic xenograft GBM compared with the negative control (1.85-fold).

#### 4.1.2. The combination of chemotherapy drugs and sensitizer

In addition to the combined use of chemotherapeutic drugs, the combination therapy with chemotherapy/radiotherapy and sensitizer can also improve the therapeutic effect on brain tumor and prevent drug resistance. Hua et al. develop the novel angiopep-2-lipid-poly-(metronidazoles)_n_ (ALP-(MIs)_n_) hypoxic radiosensitizer-polyprodrug nanoparticles to enhance the radiosensitizing effect on gliomas (Hua et al., 2018). The nanoparticles are aggregated in glioma through specifically binding with low-density lipoprotein receptor-related protein-1 (LRP-1) highly expressed on the surface of brain microvascular endothelial cells and glioma cells. The hydrophobic P-(MIs)_n_ core encapsulating DOX is converted into hydrophilic amino groups under low oxygen conditions to mimic the oxygen-increased sensitization and provoke the release of DOX. The activity evaluation shows that ALP-(MIs)_n_/DOX can effectively accumulate in the glioma after systemic administration in vivo, which shows a significant radiosensitizing effect for glioma treatment. The median survival time for mice treated with LP-(MIs)_25_+RT (61 days) and ALP-(MIs)_48_+RT (63 days) were longer than those of PBS, PBS + RT and AL-PLGA + RT, suggesting that ALP-(MIs)_25_ and ALP-(MIs)_48_ improved the efficacy of the radiotherapy. Similarly, Lam et al. develop transferrin-functionalized nanoparticles (TF-NP) to co-deliver temozolomide and chemotherapy sensitizer JQ1 (a bromine domain inhibitor), which can effectively enhance chemotherapy-induced DNA damage and cytotoxicity (Lam et al., 2018). Treatment of tumor-bearing mice with TF-NP loaded with TMZ and the JQ1 leads to increased DNA damage and apoptosis that correlates with a 1.5- to 2-fold decrease in tumor burden and corresponding increase in survival compared to equivalent free-drug dosing.

In addition to combining with sensitizers, introducing photosensitizers and thermosensitive agents can also improve the effect. Lu et al. developed a disulfide bond-conjugated prodrug polymer consisting of camptothecin (CPT) and polyethylene glycol (PEG) with further modification of iRGD peptide. The polymer could self-assemble into nanosized polymeric micelles and load with photosensitizer IR780 for combination therapy. Interestingly, conjugation of iRGD on the surface of micelles obviously enhances the ability to cross the BBB and target glioma cells, which displays a better tumor killing ability (Lu et al., 2020). CPT-S-S-PEG-iRGD@IR780 micelles combined chemotherapy with photodynamic therapy (PDT) showed the longest median survival time (49 days), while PBS, CPT, CPC@IR780 micelles and CPD@IR780 micelles treatments achieved the median survival times of 29, 31, 31 and 38 days, respectively. In our previous study, we prepared the biomimetic nanoparticles (ICG/PTX@RGE-EV) co-loading indocyanine green (ICG) and PTX by modifying Neuropilin-1 targeting peptide (RGE) on the extracellular vesicles (EV) membrane. ICG/PTX@RGE-EV shows good photothermal properties and promotion of PTX release from EVs, when stimulated by 808-nm laser light. Then, they target U251 cells, with activation of the Caspase-3 pathway and elevated apoptosis, which increases the median survival of glioma mice (Wang et al., 2021). What’s more, the significantly reduced tumor volume was observed in mice following targeting combined therapy (ICG/PTX@RGE-EV + NIR) compared with mice treated with chemotherapy, (PTX@RGE-EV), hyperthermia (ICG@RGEEV + NIR), or non-targeted chemotherapy-hyperthermia (ICG/PTX@EV + NIR).

#### 4.1.3. The combination of other small molecule chemical drugs

Antitumor drugs achieve the treatment of tumors through a variety of mechanisms. In addition to directly inducing tumor cell apoptosis, they can also inhibit angiogenesis, regulate tumor autophagy, reshape the tumor microenvironment, and regulate tumor related signal pathways. Lakkadwala et al. develop a dual-functional liposome, which is modified with Tf to target the brain endothelial cells and glioblastoma cells. The liposome is also decorated with CPP (Pen) to promote the transport of DOX and erlotinib across the BBB to glioblastoma tumor (Lakkadwala et al., 2019). The biodistribution of Tf-Pen liposomes demonstrated 12- and 3.3- fold increase in DOX and erlotinib accumulation in mice brain, respectively compared to free drugs. In addition, Tf-Pen liposomes showed excellent antitumor efficacy by regressing ∼90% of tumor in mice brain with significant increase in the median survival time (36 days) along with no toxicity.

Glioblastoma (GBM) treatment is undermined by the suppressive tumor immune microenvironment (TIME). Zheng et al. develop a liposome modified with α7 nicotinicacetylcholine eceptors (nAChRs)-binding peptide ^D^CDX to achieve a ‘three birds-one-stone’ delivery strategy, namely, targeting the glioma vessel endothelium, glioma cells, and tumor-associated macrophages that all overexpressing α7 nAChRs (Zheng et al., 2020). This multifunctional liposome co-encapsulates honokiol and disulfiram/copper complex to remodel the tumor metabolism and TIME through the mammalian target of rapamycin. The median survival time of the orthotopic cancer mice in the CDX-LIPO group was 27 days, which was significantly longer than that of the mice treated with PBS (9 days), free drug injections (17 days), free-drug combo (21 days), and LIPO (21 days).

Biotinylated PAMAM G3 dendrimers with BBB penetrating ability are used to load anticancer agent cyclooxygenase-2 inhibitor celecoxib and peroxisome proliferator-activated receptor γ agonist Fmoc-L-Leucine, which have a synergistic effect on glioma (Uram et al., 2019). Huang et al. develop acid-sensitive CaCO_3_/TPGS nanoparticles (ICG-PDA-TPZ NPs), modified with RGD peptide (Huang et al., 2019). The nanoparticles encapsulate near-infrared photosensitizer ICG, photothermal conversion agent polydopamine (PDA), and tirapazamine (TPZ), which have the synergistic treatment for brain tumors through chemo-photodynamic and photothermal therapy. In addition, the multifunctional NDDS used for the co-delivery of cobstatin-A4 (anti-angiogenesis agent) and DOX, PTX and melittin, PTX and artemether has also made good progress in the treatment of glioma (Gao et al., 2014; Li et al., 2014; Wang et al., 2019).

### 4.2. The combination of chemotherapy and chemodynamic therapy

The ROS, such as hydrogen peroxide, hydroxyl radical, superoxide anion radical and singlet oxygen, etc, are widely present in mammalian cells. When the content exceeds the tolerance value of cells, it will induce cell necrosis and apoptosis (Trachootham et al., 2009). Chemodynamic therapy (CDT), first proposed by Shi and coworkers in 2016, is an emerging cancer therapeutic method (Zhang et al., 2016). It uses various transition metal ions, such as Fe^2+^, Mn^2+^ and Cu^+^, to catalyze H_2_O_2_ decomposition in the cancer region. It has emerged as an efficient strategy for cancer treatment utilizing Fenton or Fenton-like reactions to destroy cancer cells by converting endogenous H_2_O_2_ into highly toxic reactive oxygen species (Tang et al., 2019). However, the in vivo therapeutic outcomes are highly dependent on the endogenous H_2_O_2_ amount, which is the power source for Fenton-like reactions (Ren et al., 2020).

In recent years multifunctional nano-delivery systems have made good progress in combination of chemotherapy and chemodynamic therapy for brain tumors. It has been found that a combination of nanoparticles can efficiently cross the blood-brain barrier and precisely target glioblastoma to inhibit cancer cells through chemotherapy and chemodynamic therapy, achieving excellent anti-cancer efficacy (Pan et al., 2022). There is currently research into the development of theranostic nanodrug (iRPPA@TMZ/MnO) where the presence of iRGD provides the nanodrug with a high ability to cross the BBB and penetrate the tumor tissue (Tan et al., 2020). Upon accumulation in glioma, the nanodrug responds to the tumor microenvironment with the simultaneous release of TMZ, Mn^2+^ and O_2_. The released TMZ and Mn^2+^ provide significant benefits for glioma growth inhibition through the synergistic anti-cancer effects of chemo-chemodynamic therapy. In addition, the generated O_2_ in situ reduces tumor hypoxia and enhances the therapeutic effect of chemotherapy/chemotherapy kinetics on glioma. And the in vivo anti-GBM efficacy results suggested that CuFeSe2-LOD@Lipo-CM + NIR group had a remarkable tumor inhibition rate of 84.9% which was significantly higher than those of CuFeSe2-LOD@Lipo-CM group (54.8%) and CuFeSe2-LOD@Lipo + NIR group (61.2%).

### 4.3. The combination of chemotherapy and immunization therapy

The immune system is one of the key components of TME and plays an important role in the occurrence and development of tumors. However, due to the ‘immune privilege’ of CNS, the current immunotherapy has not been clinically proven to significantly improve the survival rate of brain tumor patients. In recent years, studies have found a small number of immune cells (including T cells) in the choroid plexus matrix, cerebrospinal fluid, subarachnoid space and perivascular space, proving that there is indeed active surveillance in the CNS (Ratnam et al., 2019). This indicates that immunotherapy is important in the treatment of brain tumors.

#### 4.3.1. Combination chemotherapy with immune checkpoint inhibitors

In the immune system, immune checkpoint inhibitors are responsible for negatively regulating the activation of T lymphocytes, thereby limiting the over activation of the immune system and maintaining immune homeostasis. However, the tumors prefer to escape the clearance of the immune system by utilizing the immune checkpoints. The checkpoints, such as the over-expressed cytotoxic T lymphocyte associated antigen 4 (CTLA-4) and programmed death ligand-1 (PD-L1), hinder the recognition of tumor cells by T cells. Blocking immune checkpoints is the most effective approach in immunotherapy. The inhibition of CTLA-4 on T cells can be alleviated by using CTLA-4 molecular inhibitors or CTLA-4 monoclonal antibodies. Similarly, using PD-1 or its ligand PD-L1 to selectively block the binding between tumor cells and T cells can also promote T cells to recognize and eliminate cancer cells (Topalian et al., 2012). Although many clinical trials using immune checkpoint blocking therapy to fight against glioblastoma, the results are unsatisfactory due to the poor permeability across the BBB into the tumor. Therefore, the combination therapy based on multifunctional NDDS plays an important role in immune checkpoint blocking therapy.

Indoleamine 2,3-dioxygenase (IDO) is an immune checkpoint receptor produced by tumor cells, macrophages, and dendritic cells (DCs) within draining lymph nodes and the tumor microenvironment. 1-methyltryptophan (1MT), an IDO-specific competitive inhibitor, not only activates effector CD8^+^ T cells and inhibits immunosuppressive regulatory CD4^+^ T cells, but also activates DCs to increase antigen presentation. Kuang et al. discover iRGD-modified nanoparticles to simultaneously deliver DOX and 1MT into glioma. The nanoparticles show the capability of penetrating through BBB into the tumor, and significantly improve the accumulation of drugs in brain tumors with minimal side effects (Kuang et al., 2018). Meng et al. propose a combination therapy targeting BBB regulation and microenvironment amelioration (Meng et al., 2021). Firstly, biomimetic nanovesicles are designed to achieve targeted regulation using biomimetic technology with favorable biocompatibility and long circulation. They encapsulate an appropriate ratio of perfluorocarbon (PF) and the A_2A_R agonist, 5′-(Nethylcarboxamido)adenosine (NECA), in the red-blood-cell membrane to form BBB-regulating nanovesicles. Ultrasound is performed to gasify these PFs to break the nanostructures. Subsequently, the NECA activate A_2A_R to induce effects on endothelial cells, which transiently increases BBB permeability. TMZ is encapsulated in manganese dioxide that attached outside with an acid-responsive material, poly(ethylene glycol)-poly(β-amino ester) to improve its stability in circulation. Manganese dioxide could react with overexpressed H_2_O_2_ to produce oxygen to improve the hypoxic microenvironment. Following, the encapsulated TMZ is released. Combined with radiotherapy, more PD-L1 antibodies enter glioblastoma tissues and release the immune brake to initiate tumor-specific immune responses, so as to achieve enhanced therapeutic efficiencies of chemoradiation and immune therapy.

#### 4.3.2. Targeting tumor-associated immune cells

Tumor-associated macrophages (TAM) are the main constituents of the tumor microenvironment. These cells are usually derived from monocyte precursors of the CNS with an M2-like phenotype, which is unfavorable for the immune system to detect and kill tumor cells (Vidyarthi et al., 2019). Transforming macrophage phenotype from anti-inflammatory M2 (TAM2, immunosuppression) to pro-inflammatory M1 (TAM1, anti-tumor) phenotype not only relieves immunosuppression and triggers cytotoxic T-cell immunity, but also enhances the chemotherapy efficacy improves the prognosis of patients, and prolongs the survival time. Zhao et al. prepare the albumin nanoparticles modified with dual ligands, a TfR-binding peptide T12 and mannose (Zhao et al., 2018). The nanoparticles can efficiently pass through the BBB mediated by TfR and albumin-binding receptor SPARC that were overexpressed in both the BBB and glioma cells, thus achieving biomimetic delivery to glioma. The group given the T12/Man-BSA NPs displayed the longest survival time, with a median survival time of 42 days, compared to 32 days for the Man-BSA NP group and 28 days for the BSA NP group. Through the co-delivery of disulfiram/copper complex and regorafenib, the system efficiently inhibits the glioma cell proliferation and successfully ‘re-educated’ the protumor TAM2 toward antitumor TAM1.

### 4.4. The combination of chemotherapy and gene therapy

Within the last decade, researchers have paid close attention to the gene therapy, which is considered to be one of the most promising methods to treat cancer. Nucleic acid-based drugs, such as siRNA, miRNA, mRNA, DNA and CRISPR/Cas9, are a new class of highly specific anticancer drugs, which play the anti-tumor role in the cytoplasm or nucleus of cancer cells (Peng et al., 2020).

At present, fifteen nucleic acid drugs developed all over the world, including ten antisense nucleic acid (ASOs) drugs, four small interfering RNA (siRNA) drugs, and one nucleic acid aptamer. In addition, there are two other mRNA drugs approved as COVID-19 vaccines. However, there are no nucleic acid drugs approved for brain tumors. The application of nucleic acid drugs on brain tumors is a new field, and the combination with other drugs is also under preclinical research.

VEGF is a key regulator of tumor angiogenesis, and RNAi interference therapy can down-regulate the expression of VEGF through siRNA. Wen et al. develop angiopep-2 (AP)-modified redox-responsive nanoparticles (Ap-CSssSA/P/R) to co-deliver siVEGF and PTX (Wen et al., 2020). In vitro and in vivo Ap-CSssSA/P/R complexes showed an excellent silencing effect of VEGF gene, and complexes via LRP1-mediated targeting delivery exerted a higher neovascularization inhibition, compared to naked PTX-loaded nanoparticles. An angiopep-2 (A2)-modified cationic lipidpoly (lactic-co-glycolic acid) (PLGA) nanoparticle (A2-N) is developed by Ye et al. that can release gefitinib (Ge) and GOLPH3 siRNA (siGOLPH3) upon entering glioma cells, thus serving as a combinatorial anti-tumor therapy (Ye et al., 2019). The released siGOLPH3 effectively silences GOLPH3 mRNA expression and further promotes EGFR and pEGFR degradation, and Ge also markedly inhibits EGFR signaling. The median survival time of mice treated with A2-N/Ge/siGOLPH3 was 45 days, longer than that of the other groups. These researches confirm the feasibility of combined anti-angiogenesis and pro-apoptotic therapy for brain tumors.

Cancer stem cells are a subset of tumor cells with high self-renewal and stem cell properties. Traditional therapeutic drugs can target and eliminate tumor cells, while they lack effectiveness on tumor stem cells and are prone to recurrence. Developing new therapeutic strategies that selectively target tumor stem cells to improve efficacy has become a research hotspot in recent years (Bhargav et al., 2020). Sun et al. prepare the cationic liposomes (DP-CLPs) loaded with survivin siRNA and paclitaxel (DP-CLPs-PTX-siRNA) modified with a low-density lipoprotein receptor-related protein and an RNA aptamer bound CD133 (Sun et al., 2018). The liposome displays durable ability to target glioma cells and brain microvascular endothelial cells (BCECs) and to deliver drugs (PTX/siRNA) to CD133^+^ glioma stem cells, which exhibits great potential for targeted imaging and therapy of brain glioma stem cells. The tumor size at 19 days of the nude mice in situ implanted by CD133^+^+DP-CLPs-PTX-survivin siRNA were much more significantly decreased compared with the group of CD133^+^+PTX, CD133^+^+CLPs-PTX-survivin siRNA and the control.

## 5. Conclusions

Brain tumor is one of the most complex and lethal tumors. The challenges in treatment mainly include the BBB, BBTB, the heterogeneity and invasiveness of brain tumor cells, immune escape, tumor stem cells and tumor microenvironment. To overcome the above obstacles, it is urgently to design the multi-functional NDDS for the delivery of drugs. Compared with the single drug administration, the combination therapy can synergistically enhance the efficacy, further reduce toxic and side effects and decrease the recurrence rate. The multifunctional NDDS that target brain tumors usually has the following characteristics: the suitable physicochemical property to improve the stability in blood circulation; the ligand modification to facilitate the penetration across the BBB and further enter into tumor cells; the stimulation-response groups to promote the delivery and controlled release of drugs in cells; the modification that targeting organelles to contribute to the precision delivery to the target sites. This paper also summarizes the research progress of multifunctional NDDS in the combination therapy of brain tumors from the following sections, the combination of chemotherapy drugs, chemotherapy-chemodynamic combination therapy, chemotherapy-immunization combination therapy, and chemotherapy-gene combination therapy.

In addition to the design strategies mentioned above, the following points should be considered when designing multifunctional NDDS for combination therapy: (1) the potential neurotoxicity of nano preparations, (2) the leakage of drugs during transportation, (3) the shielding effect of the protein corona, (4) the off-target effect. Therefore, when designing brain tumor targeting NDDS, it is necessary to reasonably select the target and combined drugs, rationally design the delivery system according to the molecular mechanism, develop new nontoxic or low-toxic materials, promote the delivery and release of drugs in brain, improve the production process, pay attention to personalized administration and to achieve precise treatment. With the continuous understanding of the physiological structure of brain tumors, the discovery of new targets, the development of anti-tumor drugs, the progress of materials science and nanotechnology, and the maturity of emerging therapies such as immunotherapy, gene therapy, cell therapy, etc., the combination therapy based on multifunctional NDDS will gradually move from theory to practice in the treatment of brain tumors.
